# Cell-Cell Fusion Mediated by Viruses and HERV-Derived Fusogens in Cancer Initiation and Progression

**DOI:** 10.3390/cancers13215363

**Published:** 2021-10-26

**Authors:** Thomas Dittmar, Julian Weiler, Tianjiao Luo, Ralf Hass

**Affiliations:** 1 Institute of Immunology, Center for Biomedical Education and Research (ZBAF), Witten/Herdecke University, 58448 Witten, Germany; julian.weiler@uni-wh.de; 2 Biochemistry and Tumor Biology Laboratory, Department of Obstetrics and Gynecology, Hannover Medical School, 30625 Hannover, Germany; luo_tj@yeah.net

**Keywords:** cell-cell fusion, syncytia formation, viruses, cancer

## Abstract

**Simple Summary:**

Even though it is known that (cancer) cells can fuse, it is still less understood how (cancer) cells merge their plasma membranes, thereby giving rise to bi- and multinucleated hybrid cells. Cell-cell fusion is an energy-dependent process and so-called fusogens are a crucial type of membrane-bound proteins, which are mandatory for overcoming plasma membrane hybridization with associated energetic barriers. Viruses and fusogens of human endogenous retroviral elements are a natural reservoir of fusogenic particles and proteins that could cause bi- and multinucleation of cancer cells. Likewise, multinucleated giant cancer cells have been found in several cancers caused by oncogenic viruses suggesting a possible correlation between viruses and fusogens of human endogenous retroviral origin in cancer cell fusion.

**Abstract:**

Cell fusion is a well-known, but still scarcely understood biological phenomenon, which might play a role in cancer initiation, progression and formation of metastases. Although the merging of two (cancer) cells appears simple, the entire process is highly complex, energy-dependent and tightly regulated. Among cell fusion-inducing and -regulating factors, so-called fusogens have been identified as a specific type of proteins that are indispensable for overcoming fusion-associated energetic barriers and final merging of plasma membranes. About 8% of the human genome is of retroviral origin and some well-known fusogens, such as syncytin-1, are expressed by human (cancer) cells. Likewise, enveloped viruses can enable and facilitate cell fusion due to evolutionarily optimized fusogens, and are also capable to induce bi- and multinucleation underlining their fusion capacity. Moreover, multinucleated giant cancer cells have been found in tumors derived from oncogenic viruses. Accordingly, a potential correlation between viruses and fusogens of human endogenous retroviral origin in cancer cell fusion will be summarized in this review.

## 1. Introduction

Cell fusion represents a fundamental biological mechanism, which is mandatory for physiological processes such as fertilization, placentation, myogenesis, osteoclastogenesis and wound healing/tissue repair [1,2,3,4,5,6,7]. Likewise, cell fusion plays an important role during pathophysiological conditions including tumor development. Thus, cancer cell fusion with macrophages [8,9,10,11] or mesenchymal stroma-/stem-like cells [12,13,14] can result in tumor reduction [15], tumor promotion [16], or tumor dormancy [17,18].

Fusion also includes infection of host cells with enveloped viruses [19] and tumor development [20,21]. Even though the process of cell fusion appears phenomenologically simple, like two merging soap bubbles, it is tightly regulated whereby various molecular processes remain to be elucidated [22].

**Figure 1:**
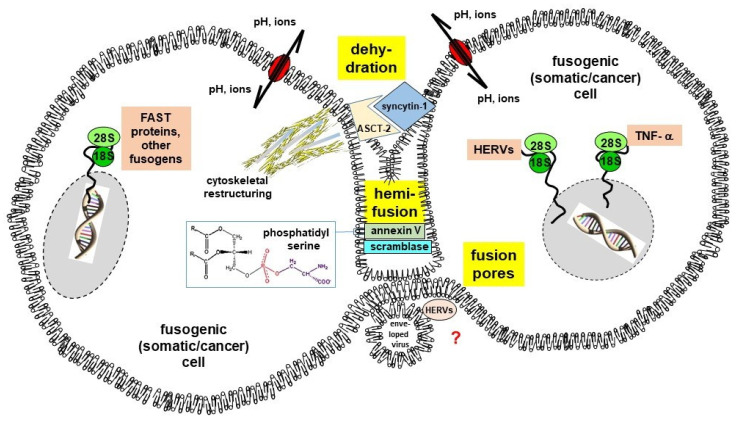
A hypothetic model of potential virus-mediated stepwise (cancer) cell fusion suggests the formation of diaphragm intermediates by the initial merging of the outer lipid layers of adjacent plasma membranes as hemifusion. Expression of fusogenic factors, e.g., syncytin-1 and the corresponding receptor alanine, serine, and cysteine selective transporter-2 (ASCT-2), together with other viral fusogens such as human endogenous retroviruses (HERV) proteins and further cell fusion inducing factors, such as tumor necrosis factor-α (TNF-α), is required within the cellular fusion partners. Concomitantly, intracellular restructuring of actin cytoskeletal proteins together with an ion gradient and low pH provide a fusion-permissive microenvironment. As a prerequisite, the plasma membranes of the somatic or cancer cells fusion partners in cooperation with the membrane of enveloped viruses have to be localized in close proximity whereby extension of membrane protrusions as lamellipodia can form local fusion pores. Whereas viruses can act as a linker for bridging fusogenic cell membranes the precise molecular role of enveloped viruses to contribute to outer membrane opening, formation of an intermediate hemifusion state, and finally the opening of the inner membrane lipid layers remains enigmatic. Among the various membrane lipids phosphatidylserine (PS) plays an important role in altering the inner lipid membrane structures to enable and finalize the fusion process. Thereby, PS interacts with associated proteins such as annexin V, scramblases, and various cytoskeletal components.

Cell-cell fusion and internalization of enveloped virus content as part of a virus-cell fusion represent multi-step processes that could be subdivided into discrete steps, such as (i) priming the prefusion state, (ii) tight binding to the target membrane, and (iii) additional intermediates between the prefusion and postfusion state [19]. The term “priming the prefusion state” indicates that per se non-fusogenic cells have to be converted first into a fusogenic state before they can hybridize with other cells [3,5,8,23]. Likewise, several pathogenic viruses, such as avian influenza virus, HIV-1, measles virus, respiratory syncytial virus (RSV), Newcastle Disease virus, Ebola Virus and even SARS-CoV-2 must be cleaved by furin or furin-like proteases to become fully activated and to be able to infect cells [24,25]. Another trigger could be a low pH that leads to a conformation change and release of the fusion peptide/loops which then could penetrate into the host cell membrane [4,5,19,26]. The term “tight binding to the target membrane” is self-explanatory since fusion requires a tight cell-cell/virus-cell contact, whereby the two plasma membranes are positioned at a distance not closer than ~10 nm [5]. The actual process of cell fusion by the merging of the plasma membranes is facilitated by so-called fusogens, which are mandatory for overcoming certain energetic barriers and steric formation of distinct (iii) lipid intermediates named “the hallmarks of cell-cell fusion” [5]. These are (a) dehydration of contacting membranes, whereby phospholipid heads are brought to distances of close to 0 nm, (b) hemifusion (merging of the outer monolayers) via a stalk and/or diaphragm intermediates, and (c) opening and expansion of fusion pore(s) from nanometer diameters to multiple microns [5] (Figure 1).

Although it is expected that basic thermodynamic and biophysical requirements encountered during the membrane fusion of enveloped viruses are the same as those occurring during fusion between cells [5,19] the process of cell-cell fusion appears to be more complex. In parallel to the induction of a fusogenic state, hybrid cells must adopt a non-fusogenic state (or post-fusion state) to prevent additional hybridization events, which, i.e., is accompanied by rearrangements of the mixing cytoskeleton and cytoplasms [3,5,19]. Likewise, hybrid cells could either remain in a bi- or multinucleated state, such as osteoclasts and syncytiotrophoblasts [3,8,27], or could undergo heterokaryon-to-synkaryon transition (HST)/ploidy reduction (PR) (HST/PR) [22,28,29,30,31].

**Figure 2:**
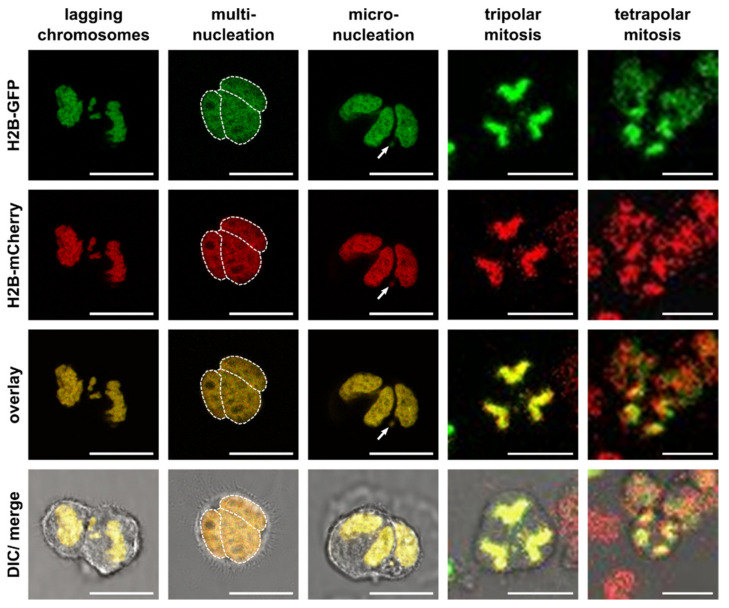
Changes in the karyotype during HST/PR by lagging chromosomes and multipolar divisions with formation of multinucleation and micronuclei and chromothripsis. Shown are representative images of hybrid cells derived from M13SV1 human breast epithelial cells that were stably transduced with pH2B-GFP (kind gift from Geoff Wahl; Addgene plasmid #11680; http://n2t.net/addgene:11680; RRID: Addgene_11680) or pH2B_mCherry_IRES_puro2 (kind gift from Daniel Gerlich; Addgene plasmid #21045; http://n2t.net/addgene:21045; RRID:Addgene_21045). The plasmids were used to trace green fluorescing GFP-expressing cells and red fluorescing mCherry expressing cells that eventually fuse by forming yellow fluorescing hybrid cells with constitutive expression of both fluorescence genes. Hybrid cells were cultured on chamber slides (ThermoFisher Scientific GmbH, Schwerte, Germany) and images and time-lapse series were recorded using a Leica TCS SP5 confocal laser scanning microscope (Leica, Wetzlar, Germany). Multiple nuclei in multinucleated cells were marked by a dashed line. The arrow points to a micronucleus. Video files of the tri- and tetrapolar cell divisions can be found in the Supplementary Materials Videos S1 and S2, respectively. Bar = 25 µm.

HST/PR is a complex and not yet fully understood mitosis-like process that requires an active cell cycle [30,31,32]. Hybrid cells contain additional copies of centrosomes (one centrosome per parental cell) and both the localization of the centrosomes and attachment of spindle fibers to chromosomes during metaphase and anaphase have an impact whether randomly mixed chromosomes will be equally segregated in a bipolar manner or missegregated due to tri- and multipolar divisions and lagging chromosomes to daughter cells (Figure 2, Videos S1 and S2). In particular, chromosome missegregation has been associated with an overall increased genomic/chromosomal instability (GCIN) due to induction of aneuploidy, multinucleation, micronuclei formation and chromothripsis [22,28,29,30,31,33,34,35,36].

In contrast, virus-infected cells have already reached a post-fusion state directly after virus-host cell membrane fusion that is commonly accompanied by virus replication and cell death. Interestingly, some members of *retroviridae* (human immunodeficiency virus), *paramyxoviridae* (Sendai virus), *poxviridae* (poxvirus), *coronaviridae* (SARS-CoV and SARS-CoV-2) [37,38,39,40,41,42,43] and *reoviridae* (Rota virus B) [19,21,26,44,45,46,47] could also lead to syncytium formation, potentially reflecting some kind of immune escape strategy to avoid capturing of free viruses by neutralizing antibodies [37,48,49,50].

The ability of cells to fuse or enveloped viruses to infect host cells is related to fusogens. Certain viral fusogens have already been characterized in detail and grouped into four classes (class I to IV) depending on their structure (see below Section 2) [19,21,26,44,51]. So far, only a small amount of proteins have been identified that facilitate cell fusion. Among them are the well-characterized human fusogens syncytin-1 and syncytin-2. These proteins facilitate the fusion of villous cytotrophoblasts, thereby giving rise to multinucleated syncytiotrophoblasts [52,53,54,55,56]. Syncytin-1 is suggested to be also involved in osteoclastogenesis [57,58] and in cancer cell fusion [59,60,61,62,63,64,65,66,67] (Table 1).

Interestingly, syncytin-1 and syncytin-2 are of retroviral origin and are related to class I viral fusogens [4,5,7,19,26]. Other known human fusogens include myomaker and myomerger involved in myogenesis [68,69,70,71] as well as Juno (oocyte) and Izumo1 (sperm) that are mandatory for fertilization [72,73,74,75]. In contrast to syncytins, these factors do not share any homologies with viral fusogens suggesting that different classes of fusogens have been developed independently during evolution. Inflammation and inflammatory cytokines, such as tumor necrosis factor-α (TNF-α), have been identified as further triggers/inducers of cell fusion [60,63,76,77,78,79,80,81,82,83] (Figure 1). Other studies revealed that spontaneous cell fusion events can also occur independently of inflammation/inflammatory cytokines [76,77,84] which underscores the heterogeneity and complexity of fusion processes.

Besides the action of fusogens, some physico-chemical conditions and membrane properties necessitate further prerequisites of cell fusion. Among these requirements is the close proximity of plasma membranes between the fusion partners accompanied by the extension of local lamellipodia-containing membrane protrusions [85]. These structures allow crosstalks between plasma membrane phospholipids, in particular phosphatidylserine (PS), PS-binding proteins, reorganization of the actin cytoskeleton and further membrane proteins (for review see: [3,4,5,23,86,87,88,89,90,91,92]). PS exposure in the outer leaflet of the membrane has been observed in virtually all fusogenic cell types, such as myoblasts, macrophages, trophoblasts, sperm and cancer cells (for review see: [4,89]). Moreover, PS exposure and signaling further plays a crucial role during membrane fusion of many enveloped viruses suggesting that PS signaling could represent a uniquely conserved module in cell-cell and viru-cell fusion (for review see: [89]) (Figure 1). Indeed, myogenesis, osteoclastogenesis and syncytization of trophoblasts were markedly impaired by masking PS, removing PS-binding annexins and inhibition/knockdown of professional phospholipid scramblases, such as TMEM16E and TMEM16F that facilitate the translocation of PS from the inner to the outer leaflet of the plasma membrane [91,93,94,95]. Similarly, entry of HIV-1 and alpha herpesvirus into host cells was markedly diminished by inhibition of scramblase activity and blockade of externalized PS [96,97] further substantiating the importance of PS in membrane fusion.

Following the increasing knowledge about cell-cell fusion, the entire process of membrane merging remains complex and requires further elucidation. In this context, virus-host cell and cell-cell fusion demonstrate some similarities that might be helpful for a better understanding of this entire process. However, molecular insights into these interactions might be limited to human fusogens, such as syncytin-1 and -2 that share homologies to class I viral fusogens. For example, myogenesis and osteoclastogenesis are not facilitated by virus-like fusogens but are rather controlled via complex multiprotein fusion machinery [4]. Therefore, common properties so far are represented by PS signaling as a more universal module in cell-cell and virus-cell fusion [89] that might apply to both, physiological and pathophysiological conditions.

Despite these findings, fusion of cancer cells is another not yet fully understood process. The hypothesis that cancer cells could fuse with, e.g., macrophages, was already postulated by the German physician, Otto Aichel, about 110 years ago [98]. Aichel postulated that aneuploidy of cancer cells could be attributed to hybridization with tumor-invading leukocytes and that the combination of extra chromosomes and the qualitative differences in chromosomes from the two cell types could lead to a metastatic phenotype [98,99]. Albeit several studies demonstrated that tumor hybrids could be detected in human cancer patients [100,101,102,103,104,105,106,107,108,109] the understanding of how the fusion of cancer cells is induced and mediated still remains less clear. This also keeps an issue for the hypothesis as to whether the fusion of two normal cells could lead to a malignant transformation of hybrid cells as suggested in several independent studies [110,111,112]. Notably, data of Duelli and colleagues indicated that this process could be attributed to virus-mediated cell-cell fusion [110,111] suggesting that viruses could act as linkers to bridge two cells, thereby causing their hybridization (Figure 1). Indeed, the first hybridoma cells were generated by Sendai virus mediated fusion of plasma cells and myeloma cells [113]. However, whether viruses are common mediators of cell-cell fusion of different cell types including cancer cells remains to be elucidated. Some viruses could lead to syncytia formation, but this has been rather assumed as some kind of immune evasion strategy and not with a malignant conversion of cells [37,48,49,50]. Likewise, a viral infection usually results in the death of the host cells either by the virus itself, induction of apoptosis or by the immune system, which also applies to virus-induced syncytia formation in oncolytic immunotherapy [114,115,116].

## 2. Viral- and Human Endogenous Retroviral-Derived Fusogens

Fusogens are indispensable for cellular hybridization to overcome fusion-associated energetic barriers [3,4,5,6,7,8,19,20] and thus, have to be expressed by fusion competent cells. Whereas cell fusion could occur in the presence or absence of inflammation [60,63,76,77,78,79,80,81,82,83,84] it can be further concluded that the presence of distinct fusogens and associated relevant factors is either induced or constitutively expressed at basal expression levels.

Besides some extensively characterized human fusogens such as syncytin-1 and -2 [52,53,54,55,56], myomerger, myomaker, actin remodeling proteins [68,70,71,117,118,119,120] as well as Izumo1 and Juno [72,73,74,75], viral fusogens or human endogenous retroviruses (HERVs) also display properties for fusion and synyctia formation capacity associated with a possible role in carcinogenesis and tumor progression.

### 2.1. Viral Fusogens

Enveloped viruses exhibit predominant capabilities to confer cell fusion due to evolutionary optimized fusogens and cell fusion strategies (for review see: [19,21,26,44,51]) (Figure 1). Depending on their structure, virus types and accompanying viral fusogens are subdivided into four classes (Table 2).

Class I viral fusogens are α-helix-rich prefusion trimers that arise from coiled-coil structures. Fusion is mediated by the insertion of hydrophobic fusion peptides or loops into membranes concomitant with refolding into postfusion trimers [19,26]. In contrast, class II viral fusogens are β-sheet-rich structures with a transition from dimers to trimers during fusion. These molecules also insert hydrophobic fusion loops into membranes ending in postfusion trimers [19,26]. Class III viral fusogens are trimers composed of both α-helices and β-sheets. In accordance with and in combination with class I and class II properties these viral fusogens insert hydrophobic fusion loops into membranes and form post-fusion trimers [19,26]. Accordingly, a common characteristic of class I to III viral fusogens is represented by facilitated virus-host membrane fusion through conserved mechanisms (for review see: [19,21,26,44,51]). To exhibit their activity, class I to III viral fusogens have to be converted into a fusogenic state and structure first. Mechanisms that contribute to such conformational activation include proteolytic cleavage, receptor binding or a change in pH [4,5,19,24,25,26]. Subsequent release of the fusion peptide/loop, with penetration and incorporation into the host cell membrane promotes fusion of the outer lipid layers by forming a hemifusion state [4,5,19,26]. A following merger of the inner lipid layers opens a fusion pore whereby pore expansion finally concludes the cell-cell merging [4,5,19,26].

Alternatively, class IV viral fusogens that are also termed fusion-associated small transmembrane (FAST) proteins, are much smaller molecules (only 20 to 40 amino acids) and do not directly mediate virus-host membrane fusion [19,26]. Instead, these FAST proteins are expressed after infection and induce syncytium formation (also named multinuclear giant cells (MNGCs)) between infected cells and non-infected adjacent cells that might function as some kind of immune escape strategy to avoid capturing of free viruses by neutralizing antibodies [19,26]. Syncytium/MNGC formation is also characteristic for some members of *Retroviridae* (human immunodeficiency virus), *Paramyxoviridae* (Sendai virus), *Poxviridae* (poxvirus), *Herpesviridae* (Herpes virus) and even *coronaviridae* (SARS-CoV and SARS-CoV-2) [37,40,41,48,49,50,51]. Thereby, syncytia/MNGC formation is most likely induced by the expression of viral fusogens in the host cell membrane with subsequent cell-cell fusion [50,51,121,122,123]. This process is similar to the origin of multinuclear syncytiotrophoblasts from syncytin-1 and syncytin-2 with the formation of villous cytotrophoblasts [52,53,54,55,56].

As an example for cell-cell fusion mediated by non-enveloped viruses, Hu and colleagues demonstrated that the human papillomavirus 16 (HPV16) E5 protein is capable to induce bi-nucleated cell formation by cell-cell fusion [124,125]. For this purpose, HPV16 E5 needs to be localized within the plasma membranes to promote cell-cell fusion [125]. However, the mechanism on how this HPV16 protein mediates the merging of plasma membranes remains to be elucidated.

Interestingly, some bacteria are also capable of inducing cell fusion such as the Gram-negative bacteria *Burkholderia pseudomallei* and the related species *Burkholderia thailandensis* [126,127,128]. Both strains can relay signals for syncytium formation by cell-cell fusion that might be beneficial for access to nutrients and immune escape. Thereby, cell-cell fusion is mediated by the type VI secretion system 5 (T6SS-5), which is evolutionary, structurally and functionally related to the tail of contractile bacteriophages and the VgrG5 effector [128,129,130,131].

A recently developed novel system for viral protein-mediated delivery and fusion with target cells is represented by the paternally expressed gene 10 (PEG10) [132] This gene product of retroelements as one of the mammalian gag homologs contributes to virus-like capsid formation that may also interact with cellular plasma membranes. Thereby, PEG10 can enable the transport of RNAs such as mRNAs and miRs among others into target cells [132,133]. Accordingly, the PEG10 system can deliver cargo to cells alternative to physiological vehicles like microvesicles or exosomes [134,135]. Moreover, the studies of Segel et al. have demonstrated that the untranslated regions of PEG10 can be reprogrammed by inserting functional mRNAs of certain genes. By developing this system of selective endogenous encapsidation for cellular delivery (SEND), specific RNAs can be addressed, e.g., for therapeutic purposes [132].

### 2.2. Human Endogenous Retroviruses (HERVs)

About 8% of the human genome is of retroviral origin with more than 400,000 HERV and 240,000 mammalian apparent long terminal repeat (LTR) retrotransposons (MaLRs) copies and related sequences [136,137,138,139]. HERVs are characterized by their 5′LTR-*gag*-*pro*-*pol*-*env*-3′LTR structure, whereas MaLRs possess a rather simple 5′LTR-ORF-3′LTR structure without a *pol* gene encoding reverse transcriptase [139]. Most of these ancient remnant elements are non-functional or inactive due to mutations, deletions and/or truncations over time, however, some of them are still active and expressed in certain tissues [136,137,138,139]. Among these prominent HERV-derived proteins are syncytin-1 (HERV-W1) and -2 (HERV-FRD) that mediate the fusion of villous cytotrophoblasts during placentation [52,53,54,55,56]. Syncytins have been identified in a variety of mammals, such as mice [140], rabbits [141], carnivorans [142], ruminants [143], opossum, and kangaroo marsupials [144] indicating an evolutionary benefit of the integration and expression of retroviral elements in placental tissues. In addition to facilitating cell-cell fusion several captured *env* genes have been proposed to exhibit an immunosuppressive role that is important for preventing maternal rejection of the semi-allogenic fetus during pregnancy [139]. Syncytin-1 and -2 share structural homologies to class I viral fusogens [4,5,7,19,26] and syncytin-1 might also play a role in osteoclastogenesis [57,58] and in cancer cell-cell fusion [59,60,61,62].

In addition to syncytins (HER2V-W1, HERV-FRD) further HERV sequences have been detected in the human genome, such as HERV-H, HERV-K, HERV9, HERV-E, HERV-Fb, HERV-V and HERV3 (HERV-R) (for review see: [138,139]). Interestingly, most HERV-related regulatory elements, such as LTRs, and/or –related proteins are active and expressed in germ cells, pre-implantation embryonic cells and the placenta (for review see: [138,139,145]). For instance, elevated transcription of HERV-H was found in human embryonic stem cells (hESCs) and human-induced pluripotent stem cells (ipSCs). This gene product was suggested to be crucial for maintaining a naïve-like state [146]. HERV-H elements provide functional binding sites for several naïve pluripotency transcription factors, such as LBP9 that drives the expression of pluripotency-associated transcription factors and pluripotency-modulating long noncoding RNA [146]. Indeed, the self-renewal capacity of hESCs and ipSCs was compromised after disruption of LBP9, HERV-H and HERV-H-derived transcripts indicating that HERV-H expression could be a hallmark of naïve-like hESCs and ipSCs [146]. This is substantiated by previous work demonstrating expression of HERV-K RNA and protein in undifferentiated but not in differentiated hESCs and ipSCs [147]. HERV-K expression was regulated by DNA hypomethylation at LTR elements together with transactivation of the stemness marker Oct4 [148]. Other studies revealed that HERV-K and HERV-H transposable elements significantly contributed to chromatin opening during human embryonic genome activation and have been identified as KLF-stimulated enhancers in naïve hESCs [149]. Interestingly, data of Göke et al. revealed a stage-specific expression of HERV elements during early embryogenesis that are related to LTRs of different HERVs, such as LTR3B and LTR14B (oocyte to four-cell; part of HERV-K14), LTR12C (zygote to eight-cell; HERV-9), MLT2A1 (HERV-L) and LTR7B (HERV-H) (both eight-cell), LTR5_Hs (HERV-K) and LTR7B (HERV-H) (both morula) and LTR7Y (HERV-H; blastocyst) [150]. Together, these findings substantiate the important role of HERV elements as regulators of pluripotency during early embryonic development.

Interestingly, nine HERV families and elements, such as HERV-K, HERV-like, HERV-V, HERV-T, HERV-W and HER2V-F, respectively, were significantly up-regulated in both hESCs and human hematopoietic stem cells (hHSCs) [151] suggesting that certain HERV elements might also play a role in the maintenance of a stem cell state in somatic stem cells. Moreover, a differential expression of HERV families and elements was also found in malignant hematopoietic cells, such as transcriptional upregulation of HERV-E family in acute megakaryocytic and erythroid leukemia, upregulation of HERV-Fc in multiple myeloma/plasma cell leukemia, and down-regulation of HERV-K in acute myeloma [151]. Whether differential expression levels of these HERV elements might contribute to the pathogenesis of such hematopoietic disorders by cancer cell fusion is not yet clear. Nonetheless, activation of selective and specific HERV-K elements/transcripts have been identified in various cancers, such as malignant melanoma, breast, ovarian and prostate cancer (for review see: [138,139,145]). Moreover, HERV-K activation was required for expansion and maintenance of a CD133^+^ melanoma cell subpopulation with stemness features [152]. Likewise, other classes of HERV *env* mRNAs were expressed in ovarian cancer (ERV3, HERV-E and HERV-K), prostate cancer (HERV-E) [153,154], and kidney cancer [155]. In addition to cancer, HERV elements have been further associated with neurodegenerative and autoimmune diseases, such as amyotrophic lateral sclerosis (ALS), multiple sclerosis (MS), rheumatoid arthritis, psoriasis and systemic lupus erythematosus [138,139,145,156,157,158,159]. Whereas these data reflect a correlation between HERV elements and stemness features of (cancer) cells, this also likely indicates an involvement of HERV elements in various diseases including cancer.

As mentioned above, both, syncytin-1 (HERV-W1) and -2 (HERV-FRD) play a pivotal role in placentation by mediating the fusion of villous cytotrophoblasts to multinucleated syncytiotrophoblasts [52,53,54,55,56]. The fusogenic capacity of other HERV *env* elements has already been investigated, but data indicate that only syncytin-1 and syncytin-2 exhibit a marked fusogenic capacity. Sugimoto and colleagues identified a HERV-Fb1-derived protein in villous cytotrophoblasts and syncytiotrophoblasts, named suppressyn, which inhibits cell-cell fusion through binding to the syncytin-1 receptor ASCT-2 [160]. Expression of ERV3-1 likely promoted fusion of BeWo choriocarcinoma cells [161], but it remains to be elucidated whether ERV3-1 is also capable to mediate the merging of other cell types. Fusogenic properties have also been postulated for the HERV-K *env* gene, which is expressed in villous and extravillous cytotrophoblast cells of the human placenta [162]. Cells could be infected with a recombinant vesicular stomatitis virus encoding HERV-K *env* (VSV-HERVK) pseudovirus [163]. Thereby, HERV-K *env* sequences bound heparin and promoted acidic pH-triggered fusion [163]. However, a distinct role of HERV-K *env* in cell-cell fusion is less clear. This also applies to HERV-E *env* proteins expressed in placenta and putatively affecting trophoblast fusion [164].

## 3. Induction of Cell-Cell Fusion by Virus-Derived Fusogens and Putative Correlation to Tumors

Fusogenic capacities of enveloped viruses and virus-related/derived proteins can stimulate the formation of heterokaryons, cell hybrids, and syncytia. However, these viruses or at least virus-derived fusogens may also be linked to cell-cell fusion and tumor development [21,165] (Figure 1). In addition to its putative role in carcinogenesis cell-cell fusion has been further associated with tumor progression. Indeed, a plethora of studies demonstrated that homotypic (tumor cell × tumor cell) and heterotypic (tumor cell × normal cell) fusion events could give rise to hybrids exhibiting an increased metastatic capacity, an enhanced drug resistance, or even cancer stem/initiating cell properties (for review see: [99,166,167,168,169,170,171,172]). In any case, despite the increasing knowledge about the impact of cell-cell fusion in tumor progression only a few proteins/phospholipids and conditions have been identified so far, which trigger the hybridization of cancer cells.

### 3.1. Syncytin-1

Several studies have demonstrated that cancer cell fusion is facilitated by syncytin-1 (HERV-W1) [59,60,61,62,63,64,65,66] (Table 1). For instance, the hybridization of human breast cancer cells with endothelial cells was mediated by syncytin-1 [62]. Syncytin-1 expression was found in about 38% of breast tumor specimens [62] that was surprisingly associated with increased recurrence-free survival [65]. In contrast, syncytin-1 expression levels and syncytin-mediated cell-cell fusion were rather correlated to disease progression in urothelial cell carcinoma [59], endometrial carcinoma and pre-stages [61], colorectal cancer [64] and prostate cancer [66]. It remains to be elucidated why cancer cells express high levels of syncytin-1. Yan and colleagues demonstrated that syncytin-1 expression in oral squamous carcinoma cells and ASCT-2 expression in endothelial cells was induced by TNF-α [60], substantiating the well-known correlation of inflammation/inflammatory cytokines as inducers of cell-cell fusion [60,63,76,77,78,79,80,81,82,83]. Constitutive basal expression of syncytin-1 protein was detectable in human MCF-7 and MDA-MB-231 breast cancer cells [62], human SCC-9 squamous carcinoma cells, MG-63 osteocarcinoma cells, Hela cells, and human umbilical vein endothelial cells [60]. Although these proteins are mandatory for overcoming energetic barriers further factors are required for successful cell fusion. As already indicated, PS signaling seems to be a uniquely conserved signaling module in cell fusion [89]. The choriocarcinoma cell line BeWo is commonly used as a model of trophoblast differentiation and cell fusion could be induced by forskolin treatment and up-regulation of syncytin-1 [161,173]. Moreover, Zhang and colleagues showed that the lipid scramblase TMEM16F, which facilitates the translocation of PS from the inner to the outer leaflet of the plasma membrane, is essential for trophoblast fusion [95]. Placentas of TMEM16F knockout mice exhibited deficiency in trophoblast syncytization and aberrant placenta development concomitant with perinatal lethality [95], supporting the relevance of PS signaling in trophoblast fusion. In addition, the placental-specific high temperature requirement factor A 4 (HtrA4) has been identified as another cell fusion associated protein since knockout of this serine protease in BeWo cells failed to undergo forskolin-induced multinucleation [174]. However, the precise role of HtrA4 in cancer cell fusion remains unclear. In any case, PS signaling and syncytin-1 expression can contribute to cancer cell-cell fusion [66,175].

Human prostate cancer cells became fusiogenic after co-cultivation with muscle cells due to an IL-4 and IL-13 induced up-regulation of syncytin-1 and annexin A5 [66]. SiRNA mediated knock-down of annexin A5 expression and likewise blockade of syncytin-1 by a synthetic peptide or shRNA markedly impaired the generation of multinucleated PC3 cells and PC3 × muscle cell heterokaryons [66] supporting the requirement of syncytin-1 and annexin A5 in prostate cancer cell fusion.

Homotypic and heterotypic hybridization of human cancer cells can be facilitated by syncytin-1 together with PS signaling (Figure 1). Albeit syncytin-1 was reported to improve the prognosis of breast cancer patients [65], most data from other carcinoma types rather indicated a relationship between syncyctin-1 and tumor progression [59,61,64,66]. These findings also suggested that fusion of cancer cells in general develops a more malignant phenotype with progression of metastatic lesions [16,20,22,99,167,169,170,172]. Further involvement of syncytin-1 in the fusion of two non-transformed cells undergoing a malignant conversion has not yet been reported and, hence, remains ambiguous.

### 3.2. Unclear Role of Other HERV Elements in Tumor Development

Various endogenous HERV elements have been associated with tumor progression, whereby HERV-K has been most extensively studied (for review see: [138,139,145,176]). Briefly, mRNA and protein levels of HERV-K elements have been found in various carcinomas, such as melanoma cell lines and tissues [177,178], breast cancer [179,180,181,182], teratocarcinoma [183,184], germ cell tumors [185], prostate cancer cell lines [181,186], ovarian cancer [154], and renal cancer [187]. Moreover, specific antibodies against the HERV-K *env* protein have been detected in breast cancer and germ cell tumors [182,185] indicating that an adaptive immune response evolved against this viral protein. Additionally, *env* protein expression of HERV-E and ERV3 was detectable in ovarian cancer [154]. Although fusogenic properties have been discussed for these proteins [163,164], a potential contribution of HERV-K *env* and HERV-E *env* to tumor initiation by the merging of (cancer) cells is still unresolved. Nonetheless, several data revealed an implication of HERV elements expression in cancer progression as activators of multiple oncogenic signaling pathways such as Wnt/β-catenin and Ras/ERK signaling and inactivators of tumor suppressor genes (for review see: [176]). For instance, the HERV-K *env* element is a strong inducer of the Ras/RAF/MAPK/ERK1/2 pathway. This can trigger the induction of several transcription factors, such as ETV4, ETV5 and EGR1, that have been associated with cellular transformation [188]. Moreover, expression of HERV-K *env* induced epithelial to mesenchymal transition (EMT) in non-tumorigenic MCF10A human breast epithelial cells indicating that this retroviral-derived element might possess oncogenic properties [188]. This was further confirmed by shRNA mediated knockdown of HERV-K *env* expression, which blocked breast cancer cell proliferation, migration, and invasion due to inhibition of the expression of tumor-associated genes including Ras, p-RSK, and p-ERK [189]. Notably, HERV-K *env* knockdown also attenuated the ability of breast cancer cells to form tumors and to metastasize [189]. Conversely, overexpression of HERV-K env in shRNAenv knockdown breast cancer cells restored Ras/RAF/MAPK/ERK1/2 signaling concomitant with the reversion of reductions in migration and invasion [189]. Interestingly, HERV-K *env* overexpression was further correlated with down-regulation of the tumor suppressor p53 [189]. Besides HERV-K env-mediated aberrant signaling in oncogenic signal transduction pathways, the HERV genomes also encodes for long non-coding RNAs, which may also facilitate breast cancer progression. In that regard, high expression levels of the HERV-derived long non-coding RNA TROJAN was found in human triple-negative breast cancer, which was additionally correlated to proliferation and invasion [190]. Of interest, TROJAN increased the degradation of the metastasis-repressing factor ZMYND8 in triple-negative breast cancer cell lines and epigenetically up-regulated metastasis-related genes in multiple cell lines [190]. Furthermore, Zhou and colleagues recently demonstrated that even syncytin-1 (HERV-W1) promoted progression and doxorubicin resistance of hepatocellular carcinoma cells via the inflammation-activated MEK/ERK pathway [191]. In agreement with breast cancer studies shRNA-mediated HERV-K *env* knockdown significantly reduced in vitro and in vivo growth rates and metastatic spreading of human pancreatic cancer cell lines concomitant with decreased expression of Ras, p-ERK, pRSK, and p-AKT [192]. Silencing of the HERV-K Np9 protein expression inhibited the growth of myeloid and lymphoblastic leukemic cells, whereas the growth of leukemia cells in vitro and in vivo was promoted by Np9 expression suggesting that Np9 might by a potent viral oncogene in human leukemia [193]. Notably, Np9 expression was further correlated to activation of ERK, AKT and Notch1 signaling pathways. Moreover, Np9 promoted up-regulation of β-catenin and increase in the overall number of leukemia stem/progenitor cells indicating that this viral oncogene represents a critical molecular switch of multiple signaling pathways regulating the growth of leukemia stem/progenitor cells [193].

In summary, an increasing body of evidence indicates an active role of HERV elements in tumor initiation and progression due to activation of multiple oncogenic signaling pathways, inhibition of tumor suppressor genes, and expression of HERV-derived long non-coding RNAs (for review see: [176]). Hence, the impact of HERV elements in cancer progression is much more complex than “transduction” of oncogenes and “insertional mutagenesis” by HERV LTRs, which has been previously suggested as the major retroviral tumorigenic mechanisms [138]. While these findings clearly indicate an involvement of HERV elements in cancer initiation and progression, the role of HERV *env* elements in cancer cell-cell fusion still remains unclear.

### 3.3. Virus-Mediated Cell-Cell Fusion and Syncytia/PGCC Formation

Multinucleated or so-called polyploid giant cancer cells (PGCCs) have been found in a variety of cancerous tissues and play a prominent role in drug resistance, invasiveness, metastasis, and stemness properties [194,195,196,197,198,199,200,201,202,203,204,205,206]. The predominant mechanisms leading to PGCC formation depend on endoreplication, mitotic slippage, cytokines failure, cell cannibalism, and cell-cell fusion (for review see: [204,207]). Likewise, different triggers of PGCC formation have been identified, such as chemotherapeutics, radiotherapy, hypoxia, oxidative stress, air pollution, UV light, hyperthermia, and oncoviruses (for review see: [204,206,207]).

Oncoviruses as PGCC inducers suggest that multinucleated cells might be derived from virus-facilitated cell merging. This is in agreement with the hypothesis of Duelli and Lazebnik suggesting an impact of virus-facilitated cell-cell fusion in cancer initiation and progression (for review see: [21]). In fact, heterokaryon formation capacity has been demonstrated for the Sendai virus, which facilitates cell merging by acting as a linker to bridge two individual cells [208]. Interestingly, the first monoclonal antibody-producing hybridomas were generated by Sendai virus-mediated fusion of plasma cells and myeloma cells [113]. Bi-nucleation of HPV16 infected cells is facilitated by the HPV16 E5 protein, but the mechanism has not yet been resolved [124,125]. HPV16 is a well-known oncogenic virus strain that could cause head and neck squamous carcinomas and among others cervical, anal, perianal, vulvar, and penile cancers [209,210]. HPV16 E6 and E7 proteins could induce genomic instability due to the generation of mitotic defects by induction of centrosome abnormalities and multipolar divisions, thereby causing aneuploidy/GCIN [211,212]. Hence, E5 mediated cell-cell fusion together with E6/E7-related centrosome abnormalities could play a role in the malignant transformation of HPV16-infected cells. Viable and highly heterogeneous hybrids were derived from Mason-Pfizer monkey virus (MPMV)-facilitated fusion of D551 fibroblasts expressing either HRAS or E1A oncogenes [111]. These findings further support the assumption of virus-facilitated cell-cell fusion as an inducer of a malignant transformation of cells. Interestingly, no MPMV-derived hybrids could be generated from wild-type D551 fibroblasts [111] possibly due to intact tumor suppressor genes/pathways that usually induce senescence or apoptosis in aneuploid (hybrid) cells [33]. Indeed, propagation of chromosome missegregation was inhibited and apoptosis was induced in aneuploid cells with intact tumor suppressors, such as p53 [213,214,215]. Conversely, the lack of tumor suppressors or their mutational inactivation was accompanied by an increased frequency and survival rate of aneuploid cells [216,217,218].

In addition to HPV, further oncogenic viruses could induce bi- and multinucleation, such as Hepatitis B and C virus, Epstein-Barr virus, Kaposi sarcoma virus and Human T-lymphotropic virus 1 (for review see [21,206,219]). However, bi-nucleation and polyploidy (and aneuploidy/GCIN) is rather induced due to a persistent expression of viral oncoproteins that leads to a dysregulation of several important cellular processes and not via cell-cell fusion [206,219]. Thus, viral oncoproteins could activate survival pathways, initiate DNA synthesis and cell cycle progression, activate proto-oncogenes, inactivate tumor suppressors and cause epigenetic modifications [206,219]. Each of these mechanisms alone or in combination could sufficiently cause replication errors, chromosome missegregation and mitotic errors, eventually leading to aneuploid and multinucleated cells including PGCCs. Altogether, cancer initiation and progression by oncogenic viruses appear to be rather attributed to non-cell-cell fusion-dependent mechanisms.

### 3.4. Oncolytic Virus-Mediated Syncytia Formation in Cancer Therapy

Oncolytic viruses have been suggested as promising candidates for cancer therapy due to their ability to specifically infect and effectively kill cancer cells. These effects are accompanied by an interruption of the immune tolerance and induction of an adaptive immune response against the cancer cells (for review see [115,116]). Limited spreading of oncolytic viruses within the tumor microenvironment remains a key challenge that could be overcome by syncytia formation via virus-mediated and/or viral fusogen-mediated cell-cell fusion (for review see [115,116]). Therefore, natural syncytia viruses such as Newcastle disease virus, Sendai virus, respiratory syncytial virus and measles virus, or so-called engineered syncytia viruses are used for oncolytic virotherapy (for review see [115,116]). Engineered syncytia viruses are generated by insertion of viral fusogens (e.g., FAST proteins, F protein of measles virus) into the backbone of non-fusogenic oncolytic viruses, such as Vesicular stomatitis virus, Herpes simplex virus or Adenovirus (for review see [115,116]). The lytic effect of syncytia formation by oncolytic viruses is multifactorial and related to direct cancer cell killing, due to e.g., induction of apoptosis, necrosis and autophagy, bystander effects of non-infected cells and non-cancer cells, and induction of an adaptive immune response (release of tumor antigens, activation of dendritic cells, cytotoxic CD8 T-cells) (for review see [115,116]). Even though apoptotic cell death and necrosis in virus-derived syncytia has been observed in several studies it became clear that induction of cell death is a highly heterogeneous process [115]. For instance, HIV and measles virus-associated apoptotic cell death is primarily due to amplification of background apoptosis in the wake of cell-to-cell fusion [220], whereas apoptosis in reovirus-induced syncytial cells is initiated due to the FAST protein-induced membrane instability [221]. In contrast, oncolytic virus-induced cell death was not prevented by pan-caspase inhibitors in hepatocellular carcinoma, non-small lung cell cancer cells, and acute myeloma cells [222,223,224] indicating that cell death was likely attributed to necrosis.

The bystander effect has been identified as an important feature in oncolytic virus therapy-induced syncytial cell death. On the one hand, the bystander effect can increase viral spreading throughout cancerous tissues, thereby improving the anti-tumoral potency of oncolytic virus therapy [225]. Likewise, the bystander effect was effective in the induction of apoptosis mediated by the HIV *env* protein [226]. HIV *env* expression in infected cells could lead to syncytia formation and activation of multiple pathways that induced mitochondrial apoptosis. Moreover, HIV *env*-mediated fusion of non-infected cells also resulted in the death of both cells, which was dependent on the mitochondrial pathway of apoptosis, but without the engagement of other multiple pathways [226].

Immunogenic cell death is another important issue in oncolytic virus therapy. In this context, the antitumoral activity of oncolytic viruses is not only related to induction of apoptosis and necrosis, but to a profound induction of a specific immune response against cancer cells. In accordance with multiple processes leading to cell death of infected cancer cells such as induction of apoptosis and necrosis, several mechanisms have been identified how oncolytic virus therapy could activate the innate and adaptive immune system. Pathogen recognition receptors (PRRs) like Toll-like receptors (TLRs) and NOD-like receptors (NLRs) are also expressed by cancer cells [227]. While this has been rather associated with tumor progression due to recognition of damage-associated molecular patterns (DAMPs) and subsequent receptor activation [227], cancer cells expressing PRRs might also recognize oncolytic virus RNA and DNA concomitant with a specific cellular response. Interestingly, virus-cell fusion specifically induced a stimulator of interferon genes (STING) response with subsequent expression of interferon-stimulated genes, in vivo recruitment of leukocytes, and potentiation of signaling via TLR7 and TLR9 [228]. Likewise, the interferon-α/β production was markedly amplified in measles virus-induced syncytial cells [229] suggesting that an antiviral cellular immune response could be fostered by cell-cell fusion. In melanoma, dying syncytia produced more so-called syncytiosomes (syncytia-derived exosomes) than normal cells, which potently loaded dendritic cells and more effectively induced a specific cytotoxic T cell response against melanoma cells expressing the specific tumor antigen gp100 [230]. Likewise, syncytia formation of human Mel888 melanoma cells reversed the suppressive effects of Mel888 on dendritic cells. Moreover, fusing melanoma cells were a more effective source for melanoma gp100 antigen presentation of dendritic cells and induction of a specific cytotoxic T cell response [231].

In summary, these findings underline the fusogenic capacities of viruses in facilitating cell-cell fusion and syncytia formation, which could be beneficial in oncolytic virus cancer therapy.

## 4. Conclusions and Future Perspectives

Cell-cell fusion has been suggested as a putative driver of tumor initiation and progression [20,99,167,168,172,232,233]. However, despite increasing knowledge the entire mechanism is still scarcely understood. The merging of two plasma membranes is a multi-step process of various proteins and fusogens that have been identified as indispensable mediators of cell-cell fusion to overcome plasma membrane merging-associated energetic barriers [3,4,5,6,7,8,19,20,89]. Enveloped and some non-enveloped viruses could induce syncytia/MNGC formation due to the expression of evolutionarily optimized fusogens [50,51,121,122,123] whereby oncogenic viruses could lead to PGCC formation [21,206,219]. About 8% of the human genome is of retroviral origin [136,137,138,139] and transcripts including *env* proteins have been identified in normal and tumorigenic tissues with fusogenic properties [138,139,145]). These effects raise the suggestion that viruses and/or fusogens of endogenous retroviral origin may represent natural mediators of cell-cell fusion during tumor initiation and progression. However, only a very few data have been published so far that support this assumption.

MPMV-fusion derived D551 fibroblast hybrids demonstrated tumorigenicity and exhibited a markedly increased GCIN, but only when hybrids were derived from HRAS or E1A expressing fibroblasts [111]. Hybrids derived from wild-type D511 fibroblasts with intact tumor suppressor machinery were not viable [111] which supports the correlation of non-functional tumor suppressors and higher tolerance to aneuploidy/polyploidy [213,214,217,234]. These data likely indicate that viruses could fuse and transform cells with an impaired tumor suppressor machinery. MPMV is a retrovirus with a host range largely restricted to primates that was also detected in human and human cancer cell lines, but displayed no identified pathogenic effect [110]. Likewise, the Sendai virus is a highly transmissible and fusogenic respiratory virus in rodents, but is considered apathogenic in humans and, hence, highly suitable for oncolytic virotherapy [115,116] and vector-based vaccination strategies [235,236]. Although apathogenic and fusogenic viruses might be putative candidates for cell-cell fusion this requires some consecutive prerequisites. These include close contact, infection and uptake of viruses/viral particles from a virus/viral particle producing organisms, successful fusion of cells with a defective tumor suppressor machinery, and survival of resulting hybrids concomitant with malignant transformation. Given that each of these steps is rate-limiting, the likelihood of this sequence of events should be extremely low. Moreover, as stated above, virus-mediated fusion rather leads to the generation of multinucleated and non-viable syncytial cells, which further decreases the overall probability that viruses could cause cellular transformation by cell-cell fusion. Accordingly, the conclusion that virus-mediated hybridization might represent a common event in tumor initiation requires further evidence.

More convincing data are available for HERV elements. Expression of HERV *env* has been identified in a variety of normal and tumor tissues [138,139,145] and is associated with tumor initiation and progression due to activation of oncogenic signal cascades [188,191,192] and induction of the EMT program [189]. Hence, HERV *env* elements rather foster tumor progression in a non-cell-cell fusion-dependent manner albeit fusogenic properties have been postulated for HERV-K *env* and HERV-E *env* [163,164], but not yet validated. In that regard, it would be interesting to investigate the potential fusogenic capacities of HERV-K *env* and HERV-E *env.*

Syncytin-1 (HERV-W1) is a well-characterized fusogen of HERV origin and its involvement in cancer cell fusion has been documented in several studies [59,60,61,62,63,64,65,66] although further molecules and signals are required to conclude this process (Figure 1). Syncytin-1 expression in cancer cells is usually associated with their fusogenic capacity. However, the reason and the benefit of cancer cells to express this fusogen and to fuse with other cells is not clear. Although cell-cell fusion appears as an inefficient process Miroshnychenko et al. suggested that spontaneous somatic cell-cell fusion enables populations of cancer cells to amplify clonal heterogeneity, which may substantially accelerate a tumor’s ability to adapt to new selective pressures [237]. The majority of tumor hybrids will die in a post-hybrid selection process (PHSP) [168] and only a small population, if any, will survive. Likewise, syncytin-1 expression is not sufficient for cell-cell fusion without expression of the ASCT-2 receptor on adjacent cells [62]. Recent findings revealed that even syncytin-1 could induce an oncogenic signaling cascade in hepatocellular carcinoma cells [191], likely indicating another crucial role of this fusogen in tumor progression. In that regard, it would be interesting to investigate whether syncytin-1 induced oncogenic signaling cascades would be also active in other cancer cells and whether these could prevent tumor hybrids from cell death.

Even though viruses and HERV *env* elements appear to be a natural reservoir of fusogens that could facilitate the merging of two and more cells, their role in cancer cell fusion is still not well understood. So far, syncytin-1 is the only known and to date best characterized fusogen of HERV origin. Its impact on cancer cell fusion and tumor progression has been demonstrated in several studies. Fusogenic properties have been postulated for other HERV *env* elements, but have not yet been clearly validated. Nonetheless, their impact on tumor progression due to activation of oncogenic signaling pathways is well documented. Oncogenic viruses could cause PGCC formation, but polyploidy is predominantly induced by a persistent expression of viral oncoproteins with dysregulation of several important cellular tumor suppressors and cell cycle regulators rather than by cell-cell fusion.

Hence, despite an inherent fusogenecity, enveloped and some non-enveloped viruses most likely do not foster cancer initiation and progression by facilitating cell-cell fusion. Conversely, the impact of distinct HERV env elements on tumor progression has been demonstrated in several studies including the biological phenomenon of cell-cell fusion. In any case, the mechanism of cell-cell fusion in tumors is still scarcely understood and more research is also necessary to elucidate the impact of virus-derived fusogens in this process.

## Figures and Tables

**Table 1:** Syncytin-1 expression and mediated fusion in normal human cells and human cancer cells.

Cell Type	Fusion Partner	Kind Of Fusion	References
villous cytotrophoblasts	villous cytotrophoblasts	homotypic	[52,53,54,55,56]
osteoclasts	bi-nucleated osteoclasts	homotypic	[57,58]
breast cancer cells	endothelial cells/ mesenchymal stem/stromal cells	heterotypic	[62,65] [63]
colorectal cancer cells	colorectal cancer cells	homotypic	[64]
endometrial cancer cells	endometrial cancer cells	homotypic	[61]
oral squamous carcinoma cells	HUVEC	heterotypic	[60]
prostate cancer cells	prostate cancer cells/ muscle cells	homotypic/ heterotypic	[66]
seminoma cells		unclear	[67]
urothelial cell carcinoma cells	urothelial cell carcinoma cells	homotypic	[59]

**Table 2:** Viral fusogens.

Class	Virus Family	Virus Strain	Fusogen	Structure	References
I	*Cornaviridae*	SARS, MERS, SARS-CoV-2	Spike protein (S protein)		[19,21,26,44,51]
	*Filoviridae*	Ebola	Ebola glycoprotein (GP)	α-helix-rich	[19,21,26,44,51]
	*Orthomyxoviridae*	Influenza virus	Hemagglutinin (HA)		[19,21,26,44,51]
	*Paramyxoviridae*	Sendai virus	F glycoprotein		[19,21,26,44,51]
	*Retroviridae*	HIV	glycoprotein 41 (gp41)		[19,21,26,44,51]
II	*Alphaviridae*	Semliki Forest virus	E1 glycoprotein		[19,21,26,44,51]
	*Flaviviridae*	Dengue virus, West Nile virus, Zika virus	E glycoprotein	β-sheet-rich	[19,21,26,44,51]
	*Matonaviridae*	Rubella virus	E1 protein		[19,21,26,44,51]
III	*Baculoviridae*	Baculovirus	gp64 glycoprotein		[19,21,26,44,51]
	*Herpesviridae*	Herpes Simplex virus, Varicella Zoster virus	gB glycoprotein	α-helices and β-sheets	[19,21,26,44,51]
	*Rhaboviridae*	Vesicular Stomatitis virus; Rabies virus	G glycoprotein		[19,21,26,44,51]
IV	*Reoviridae*	Rota virus B	FAST protein	non-structural simplified domains	[19,21,26,44,47,51]

## Data Availability

The data presented in this study are available on request from the corresponding author.

## Supplementary Materials

The following are available online at https://www.mdpi.com/article/10.3390/cancers13215363/s1, Video S1: Video of a tripolar cancer cell division from Figure 2, Video S2: Video of a tetrapolar cancer cell division from Figure 2.

## Abbreviations

ASCT-2	alanine, serine, and cysteine selective transporter-2
EMT	epithelial to mesenchymal transition
FAST	fusion-associated small transmembrane
GCIN	genomic/chromosomal instability
HERV	human endogenous retrovirus
HST	heterokaryon-to-synkaryon transition
HtrA4	high temperature requirement factor A 4
hESCs	human embryonic stem cells
hHSCs	human hematopoietic stem cells
HPV	human papillomavirus
ipSCs	induced pluripotent stem cells
LTR	long terminal repeat
MaLR	mammalian apparent LTR retrotransposon
MNGCs	multinuclear giant cells
MPMV	Mason-Pfizer monkey virus
NLR	NOD-like receptor
PGCC	polyploid giant cancer cells
PR	ploidy reduction
PRR	pathogen recognition receptor
STING	stimulator of interferon genes
TNF-α	tumor necrosis factor-α
TLR	Toll-like receptor
PEG10	paternally expressed gene 10
PS	phosphatidylserine
